# Longitudinal effects of adjuvant chemotherapy and related neuropathy on health utility in stage II and III colon cancer patients: A prospective cohort study

**DOI:** 10.1002/ijc.33472

**Published:** 2021-01-25

**Authors:** Gabrielle Jongeneel, Marjolein J. E. Greuter, Felice N. van Erning, Jos W. R. Twisk, Miriam Koopman, Cornelis J. A. Punt, Geraldine R. Vink, Veerle M. H. Coupé

**Affiliations:** ^1^ Department of Epidemiology and Data Science Amsterdam UMC, VU University Amsterdam The Netherlands; ^2^ Department of Research and Development Netherlands Comprehensive Cancer Organisation (IKNL) Utrecht The Netherlands; ^3^ University Medical Center Utrecht, Utrecht University Utrecht The Netherlands; ^4^ Julius Center for Health Sciences and Primary Care University Medical Center Utrecht Utrecht The Netherlands

**Keywords:** adjuvant chemotherapy, colon cancer, longitudinal data, quality of life

## Abstract

Patient's quality of life should be included in clinical decision making regarding the administration of adjuvant chemotherapy (ACT) in stage II/III colon cancer. Therefore, quality of life, summarized as health utility (HU), was evaluated for patients treated with and without ACT. Furthermore, the role of chemotherapy–induced peripheral neuropathy (CIPN) on HU was evaluated. Patients diagnosed with stage II/III colon cancer between 2011 and 2019 and participating in the Prospective Dutch ColoRectal Cancer cohort were included (n = 914). HU scores were assessed with the EQ‐5D‐5L at baseline, 3, 6, 12, 18, and 24 months. Patients treated with ACT received mainly capecitabine and oxaliplatin (57%) or capecitabine monotherapy (40%) (average duration: 3.5 months). HU 3 to 18 months after diagnosis (potential ACT period + 12 months follow‐up) was compared between patients treated with and without ACT using a mixed model adjusted for age, sex and education level. Subsequently, the CIPN sensory, motor and autonomy scales, measured using the EORTC QLQ‐CIPN20, were independently included in the model to evaluate the impact of neuropathy. Using a mixed model, a significant difference of −0.039 (95% confidence interval: −0.062; −0.015) in HU was found between patients treated with and without ACT. Including the CIPN sensory, motor and autonomy scales decreased the difference with 0.019, 0.015 and 0.02, respectively. HU 3 to 18 months after diagnosis is significantly lower in patients treated with ACT vs without ACT. This difference is on the boundary of clinical relevance and appears to be partly related to the sensory and motor neuropathy‐related side effects of ACT.

## Abstract 2

**What's new?**

Given its potentially serious side effects, patient quality of life should be included in clinical decision‐making regarding the administration of adjuvant chemotherapy in stage II/III colon cancer. Using a mixed model which was corrected for relevant covariates, here the authors found a statistically‐significant but small decrease in HU of −0.039 during chemotherapy and the following 12 months for patients treated with adjuvant chemotherapy compared to patients without adjuvant chemotherapy. The decrease in HU, which is on the boundary of clinical relevance, appears to be partly related to sensory and motor neuropathy side effects of adjuvant chemotherapy.

**Abbreviations**
ACT: adjuvant chemotherapy
CAPOX: capecitabine combined with oxaliplatin
CI: confidence interval
CIPN: chemotherapy‐induced peripheral neuropathy
FOLFOX: fluorouracil, leucovorin and oxaliplatin
HU: health utility
MID: minimally important difference
PLCRC: Prospective Dutch Colorectal Cancer Cohort
QALYs: quality‐adjusted life years

## 1 BACKGROUND

Colon cancer is one of the most frequently occurring cancers in the Netherlands with approximately 10 000 new cases and 3750 deaths in 2018.^1^ Around 60% of newly diagnosed colon cancer patients are classified as stage II or III. The standard treatment for stage II and III colon cancer patients is surgical resection, followed by an additional treatment with adjuvant chemotherapy (ACT) for those with a high risk of recurrence, that is, stage II patients with pT4 and MSS and all stage III patients.^2^

Currently, the standard ACT regimen for high‐risk stage II and stage III patients in the Netherlands consists of 3 months capecitabine and oxaliplatin (CAPOX).^3^, ^4^, ^5^ However, the benefit in overall survival that has been demonstrated in prospective studies^2^ only concerns a subgroup of patients that cannot be identified upfront. As a result, the majority of patients do not derive any benefit from ACT, either because they are cured by surgery alone or experience a recurrence despite ACT. Treatment with CAPOX is associated with side effects such as fatigue, hand‐foot syndrome, bone marrow suppression, nausea, diarrhea and vomiting.^6^, ^7^ In addition, oxaliplatin causes neurotoxicity in approximately 30% of patients, which is acknowledged as the most severe and sometimes irreversible side effect of ACT.^3^, ^6^ Neurotoxicity means that the nerves are damaged, causing discomfort in patients' daily life.^8^ For approximately 5% of the patients, the complaints are chronic.^3^, ^8^ Given the possible severity of side effects of ACT, patients should be well informed on their decision regarding the administration of ACT.

To support decision making, policy makers often use economic evaluations, in which the value of a medical intervention is evaluated by carefully balancing the health impact of the intervention against the costs. In economic evaluations, health effects are expressed in quality‐adjusted life years (QALYs), which is a measure of disease burden, including both the quality and the quantity of life years lived. One QALY is equal to 1 year of life in perfect health. To calculate QALYs, life years lived is weighted with a health utility (HU), ranging from 0 (dead) to 1 (perfect health). Thus, health utilities are important input for economic evaluations.^9^, ^10^

However, literature data regarding the influence of ACT on HU in stage II and III colon cancer are scarce, outdated and concerns mixed populations of patients with colon and rectal cancer.^11^, ^12^, ^13^ Furthermore, there are indications that HU changes over time during the different phases in a patient's course of disease, that is, prior to, during and after ACT treatment.^14^, ^15^ The studies conducted so far are less suitable to evaluate HU over time given their retrospective nature.^11^, ^12^ In addition, given that chemotherapy‐induced peripheral neuropathy (CIPN) is a common side effect of ACT with direct consequences for patients' daily life, the degree of CIPN may influence HU among ACT‐treated patients.^8^ To our knowledge, no previous studies investigated in detail the impact of ACT on HU over time in colon cancer and assessed the impact of CIPN on HU.

Given the limited knowledge in this field, our primary aim was to investigate the longitudinal impact of ACT on patients' HU in stage II and III colon cancer patients. As a secondary aim, we evaluated the impact of CIPN on the association between ACT and HU. We used data from the Prospective Dutch Colorectal Cancer cohort (PLCRC), a nationwide registry of patients with colorectal cancer.^16^

## 2 METHODS

### 2.1 Patient population and selection

We used data from the PLCRC, which is a prospective multidisciplinary nationwide observational cohort study in the Netherlands.^16^ All colorectal cancer patients (stages I‐IV) are eligible for inclusion. After patients have given their informed consent, longitudinal clinical data are registered and patient‐reported outcome measures are collected. For the present study, 2526 questionnaires of 914 participants with an average age of 66 years and diagnosed with stage II or III colon cancer between 2011 and 2019 were available.

**FIGURE 1:**
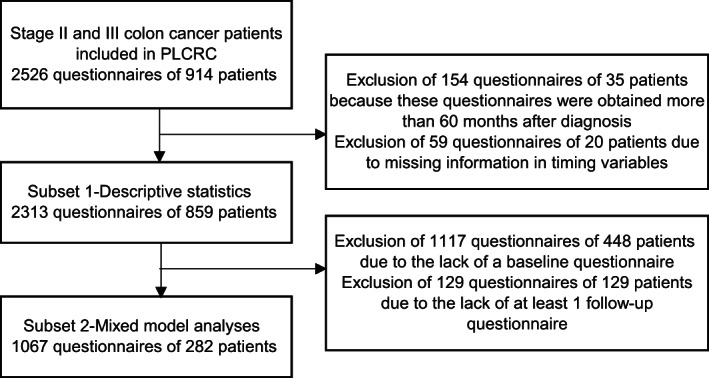
Flowchart of patient selection in Prospective Dutch Colorectal Cancer Cohort (PLCRC) cohort

Patients received a questionnaire at baseline and at 3, 6, 12, 18 and 24 months follow‐up. Patients were included at different time points after diagnosis (0‐240 months). Only 45% of the patients were included directly after diagnosis. To avoid distortion of the results by patients who were included in the cohort extremely long after diagnosis, all questionnaires filled in more than 60 months after diagnosis were excluded (Figure 1). All remaining 2313 questionnaires of 859 patients were linked to a specific time period: (a) prior to surgery, (b) after surgery and before start chemotherapy, (c) during chemotherapy, (d) first 12 months after chemotherapy and (e) more than 12 months after chemotherapy. For patients who did not receive ACT, the same time periods as in the ACT group were used. For this purpose, we defined the periods “after surgery and before chemotherapy” and “during chemotherapy” for the group without ACT based on mean time between surgery and the start of chemotherapy (1 month) and the mean duration of chemotherapy (3.5 months) in the group with ACT. Measurements for which the timing was missing were excluded (Figure 1).

Firstly, we described average HU in the defined time periods for patients with and without ACT. In this analysis, we included all measurements that were taken within 60 months after diagnosis (subset 1). Secondly, we conducted mixed model analyses to compare HU between patients treated with and without ACT during chemotherapy and the first 12 months thereafter, taking into account repeated measurements of patients and relevant covariates. To correct for differences in HU at baseline in the mixed model, only patients were included for whom at least one baseline measurement (before the start of ACT) and one follow‐up measurement was available (subset 2).^17^ A flowchart of the data is shown in Figure 1.

### 2.2 Study measures

The available data regarding administration of ACT were based on the patients' medical record. It should be noted that in 2018 the Dutch guideline recommended to limit the duration of oxaliplatin‐based ACT from 6 to 3 months for stage III patients. In 2019, this guideline adaption was made for high‐risk stage II patients.^5^ The majority of the patients (approximately 85%) were included in the cohort prior to this guideline adjustment and were therefore scheduled for a duration of 6 months of ACT.

CIPN was measured using the European Organization for Research and Treatment of Cancer Quality of Life Questionnaire Chemotherapy Induced Peripheral Neuropathy 20 (EORTC QLQ‐CIPN20).^18^ This questionnaire consists of 20 items evaluating sensory, motor and autonomic symptoms. The items were measured on a four point Likert scale ranging from 1 (not at all) to 4 (very much). Subsequently, the individual items belonging to sensory, motoric and autonomic symptoms were summarized in an average score ranging from 0 to 100 for each domain separately.^18^ For an overview of the separate items belonging to the scales see Appendix S1. The internal consistency of the scales was examined with Cronbach's alpha coefficients. In line with the literature, an internal consistency of 0.7 or higher was considered as adequate.^18^, ^19^ The Cronbach's alpha was .81, .84 and .51 respectively for the sensory, motor, and autonomy scales. Given the poor internal consistency of the two items included in the autonomy scale, we examined these two items individually.

HU was assessed with the EQ‐5D‐5L, which consists of five questions evaluating the health dimensions mobility, self‐care, usual activities, pain/discomfort, and anxiety/depression on a five‐point Likert scale ranging from 1 (no complaints) to 5 (extreme complaints).^20^ The patients' scores on these health dimensions were transformed into a utility score using the Dutch tariff.^21^

### 2.3 Descriptive patterns of HU over time

Using the patient population included in subset 1, we calculated average HU with 95% confidence intervals (CIs) for the abovementioned time periods separately for patients treated with and without ACT. When a patient had completed two or more questionnaires during one of the defined time periods, an average value for this patient was calculated before the average utility was estimated for the population.

### 2.4 Mixed‐model analyses

Linear mixed models were used to study the difference in HU during chemotherapy and the first 12 months thereafter between patients with and without ACT. Measurements in time periods “before surgery” and “after surgery and before chemotherapy” were considered as baseline measurements. In the rare case of two baseline measurements (n = 2), the two baseline measurements were averaged. Measurements in the periods “during chemotherapy” and “first 12 months after chemotherapy” are follow‐up measurements. Because of absence of either a baseline measurement or at least one follow‐up measurement, 1246 measurements of 577 patients were excluded (Figure 1). It should be noted that this large loss in number of measurements is mainly due to the fact that many of the excluded patients were included in PLCRC relatively long after primary diagnosis (more than 1 year after the start of ACT).

Firstly, we developed a crude model, which included adjuvant treatment (yes/no), baseline HU measurement and time between the start of the chemotherapy and the follow‐up measurement. Secondly, the model was adjusted for age, sex and education level, based on previous literature.^14^ This adjusted model was used to estimate the total effect of chemotherapy (yes/no) on HU. As a third step, we independently included the CIPN scores for the sensory, motor and autonomy scales in the adjusted model to evaluate the impact of neuropathy, that is, the direct effects. Note that the two items belonging to the autonomy scale were included as separate items in the mixed model, given the poor internal consistency at scale level. Correlation between repeated measurements within one individual was taken into account by using a random intercept in all analyses. The indirect effects, that is, the impact of the sensory, motor and autonomy neuropathy scales, were calculated by subtracting the direct effects from the total effect.

### 2.5 Sensitivity analyses

In the mixed models, we were not able to include disease stage as a confounder due to the strong correlation between ACT and stage (correlation .73).^17^, ^22^ Given the baseline difference in stage between the group with and without ACT, we conducted two sensitivity analyses to investigate the potential impact of stage on the association between ACT and HU. First, we conducted a propensity score matching analysis to correct for potential confounding by indication. The propensity score was estimated in a logistic regression model with ACT as dependent variable. Using a backward selection procedure the independent variables age, sex, education level, baseline HU and disease stage were evaluated as predictors for treatment. Age and stage were included in the final model. Based on the propensity score, 38 patients who received ACT were matched 1:1 to 38 patients who did not receive ACT using the R matching package.^23^ Subsequently, the mixed‐model analyses described above were repeated in the matched population.

Second, we conducted a subgroup analysis in which we only included patients for which the baseline measurement was taken during the time period “after surgery and before chemotherapy,” thereby excluding patients with a baseline measurement that was taken during the time period “before surgery.” The rationale behind this is that before surgery, patients do not know the postsurgical disease stage and the potential need for ACT. However, after surgery the disease stage is known and as a result, stage III colon cancer patients may experience more mental stress than stage II colon cancer patients after surgery and before chemotherapy. We corrected for this potential difference in mental stress by only selecting the patients with a baseline measurement in the time period “after surgery and before chemotherapy” and including the baseline HU measurement in the model.

## 3 RESULTS

### 3.1 Baseline characteristics

In subset 1, 1100 measurements of 405 colon cancer patients treated without ACT and 1113 measurements of 454 colon cancer patients treated with ACT were included (Table 1). Patients treated with ACT were younger during all defined time periods compared to patients treated without ACT. The majority of the patients were men in both treatment groups in all defined time periods. Furthermore, the majority of the patients treated without ACT had stage II disease and the majority of the patients treated with ACT had stage III disease (Table 1). Because only 1 out of the 10 comparisons between stage II and III colon cancer patients was statistically significant, namely the comparison in the no ACT group for the time period more than 12 months after chemotherapy (Appendix Table S1), we decided to further analyze stage II and III colon cancer patients together.

**TABLE 1:** Baseline characteristics of PLCRC subset 1 per defined time period

	Before surgery		After surgery and before chemotherapy		During chemotherapy		End chemotherapy—12 months after chemotherapy		More than 12 months after chemotherapy	
	Untreated (n = 110)	Treated (n = 89)	Untreated (n = 100)	Treated (n = 114)	Untreated (n = 226)	Treated (n = 206)	Untreated (n = 387)	Treated (n = 497)	Untreated (n = 277)	Treated (n = 307)
Age, median (IQR)	70 (61‐75)	64 (59‐72)	68 (59‐74)	65 (57‐71)	70 (61‐75)	66 (57‐71)	69 (61‐75)	65 (59‐71)	67 (60‐74)	65 (59‐71)
Sex, number (%)										
Men	76 (69)	63 (71)	61 (61)	56 (49)	145 (64)	110 (54)	248 (64)	284 (57)	154 (56)	187 (61)
Women	34 (31)	26 (29)	39 (39)	58 (51)	831(36)	96 (46)	139 (36)	213 (43)	123 (44)	120 (39)
Disease stage, number (%)										
II	89 (81)	10 (11)	79 (79)	8 (7)	185 (82)	21 (10)	305 (79)	47 (9)	204 (74)	36 (12)
III	21 (19)	79 (89)	21 (21)	106 (93)	41 (18)	185 (90)	82 (21)	450 (91)	73 (26)	271 (88)
Neuropathy, median (IQR)^a^										
Sensory	0 (0‐3.7)	0 (0‐3.7)	0 (0.3.7)	0 (0–3.7)	3.7 (0‐11.1)	14.8 (7.4‐25.9)	3.7 (0‐11.1)	14.8 (3.7‐29.6)	3.7 (0‐14.8)	14.8 (3.7‐25.9)
Motoric	0 (0‐4.8)	0 (0‐4.8)	4.8 (0‐9.5)	0 (0‐4.8)	0 (0‐14.3)	9.5 (4.8‐23.8)	4.8 (0‐9.5)	9.5 (0‐21.4)	4.8 (0‐9.5)	9.5 (0‐19.0)
Autonomy	0 (0‐16.7)	0 (0‐0)	0 (0‐16.7)	0 (0‐16.7)	0 (0‐0)	0 (0‐16.7)	0 (0‐16.7)	0 (0‐16.7)	0 (0‐16.7)	0 (0‐16.7)
Treatment regimen^b^										
Capecitabine monotherapy	NA	33 (37)	NA	35 (31)	NA	92 (45)	NA	228 (46)	NA	118 (38)
CAPOX	NA	55 (62)	NA	76 (66)	NA	110 (53)	NA	259 (52)	NA	131 (43)
FOLFOX	NA	1 (1)	NA	2 (2)	NA	3 (1)	NA	4 (1)	NA	4 (1)
Unknown	NA	NA	NA	1 (1)	NA	1 (1)	NA	6 (1)	NA	54 (18)

*Note*: This table shows 2313 measurements from 859 patients.

Abbreviations: CAPOX, capecitabine plus oxaliplatin; FOLFOX, fluorouracil, leucovorin and oxaliplatin; IQR, interquartile range; NA, not applicable; PLCRC, Prospective Dutch Colorectal Cancer Cohort.

^a^ Measured on a scale from 0 to 100.

^b^ Note that we reported the treatment regimen that patients will receive in the future or have received in the past in the defined time periods other than “during chemotherapy.”

In subset 2, 585 measurements of 153 patients treated without ACT and 482 measurements of 129 patients treated with ACT were included (Table 2). At baseline, the median age was lower in the ACT group (difference: 5 years). The majority of the patients treated without ACT were stage II (82%). Among patients treated with ACT the percentage of stage II patients was only 9%. All other baseline characteristics were comparable between the ACT and no ACT group. ACT consisted mostly of capecitabine plus oxaliplatin (CAPOX, 57%) and capecitabine monotherapy (40%). The average treatment duration was 3.5 months. Note that the baseline characteristics of subset 1 and subset 2 were in line with each other.

**TABLE 2:** Baseline characteristics for PLCRC subset 2, which was used for the mixed model analyses

	All (n = 282)	No chemotherapy (n = 153)	Chemotherapy (n = 129)
Age, median (IQR)	67 (61‐74)	70 (61‐75)	65 (59‐71)
Sex, number (%)			
Men	175 (62)	97 (63)	78 (60)
Women	107 (38)	56 (37)	51 (40)
Disease stage, number (%)			
II	136 (48)	125 (82)	11 (9)
III	146 (52)	28 (18)	118 (91)
Treatment regimen, number (%)			
Capecitabine monotherapy	NA	NA	52 (40)
CAPOX	NA	NA	74 (57)
FOLFOX	NA	NA	2 (2)
unknown	NA	NA	1 (1)
Average treatment duration in months, mean (SD)	NA	NA	3.5 (1.4)
Education level, number (%)			
Low	106 (38)	61 (40)	45 (35)
Moderate	75 (27)	36 (24)	39 (30)
High	97 (34)	53 (35)	44 (34)
Missing	4 (1)	3 (1)	1 (1)
Neuropathy baseline measure, median (IQR)^a^			
Sensory	0 (0‐7.4)	0 (0–7.4)	0 (0–3.7)
Motoric	0 (0–9.5)	0 (0–9.5)	0 (0–4.8)
Autonomy	0 (0‐16.7)	0 (0‐16.7)	0 (0‐16.7)
Neuropathy follow‐up measurements, median (IQR)^a^			
Sensory	7.4 (1.9‐22.2)	3.7 (0‐10.6)	18.5 (7.4‐29.6)
Motoric	7.1 (1.9‐16.7)	4.8 (0‐13.1)	11.1 (4.8‐19.0)
Autonomy	1.3 (0‐16.7)	0 (0‐12.5)	11.9 (6.7‐16.7)

Abbreviations: CAPOX, capecitabine plus oxaliplatin; FOLFOX, fluorouracil, leucovorin and oxaliplatin; IQR, interquartile range; NA, not applicable; PLCRC, Prospective Dutch Colorectal Cancer Cohort.

^a^ Measured on a scale of 0 to 100.

### 3.2 Descriptive patterns of HU over time

**FIGURE 2:**
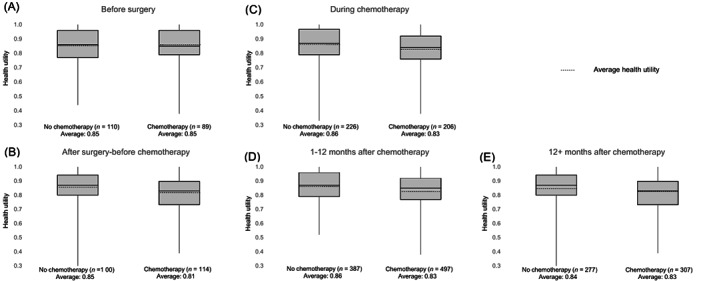
Boxplots indicating average HU, separately for with chemotherapy treated and untreated patients for the time periods before surgery, A, after surgery and before chemotherapy, B, during chemotherapy, C, 1 to 12 months after chemotherapy, D, and 12+ months after chemotherapy, E. Note that in the no adjuvant chemotherapy group, the same time points in terms of months were used as in the adjuvant chemotherapy group, to define the chemotherapy‐related time periods

In Figure 2, the HU measurements for the different time periods for patients treated with and without ACT are summarized by means of boxplots. After surgery and before start of chemotherapy we found a difference in HU of 0.04 between patients treated with ACT (average: 0.81) and those treated without (average: 0.85) (Figure 2B). We also observed a difference in average HU between the group treated with ACT (average 0.83) and the group treated without ACT (average 0.86) in the periods “during chemotherapy” and “first year after chemotherapy” (Figure 2C,D). For the time period “more than 12 months after chemotherapy” we found a small difference of 0.01 between the group treated with ACT (average 0.83) and for the group treated without ACT (average 0.84) (Figure 2E). For the time period “before surgery” average health utilities were comparable in both groups, with a value of 0.85 (Figure 2A).

### 3.3 Linear mixed‐model analyses

Table 3 provides the crude and adjusted parameter estimates of the linear mixed model in which we investigated the association between ACT and HU during chemotherapy and the first 12 months thereafter. Note that the residuals in the mixed models were normally distributed after including the HU baseline measurement. A significant difference of −0.039 (95% CI: −0.062; −0.015) in HU was found for the group treated with ACT compared to the group treated without ACT in a mixed model adjusted for age, sex and education level (total effect). Subsequently, we independently included the CIPN sensory, motor and autonomy scales to evaluate the impact of neuropathy in the association between ACT and HU, that is, the direct effects. The neuropathy scores for the sensory, motor and autonomy scales were considerably higher in the group treated with ACT compared to the group treated without ACT during follow‐up (Table 2). Including the sensory CIPN scale resulted in a coefficient for ACT of −0.020 (95% CI: −0.044; 0.003), which indicated an indirect effect of 0.019. Furthermore, the association between ACT and HU over time was no longer significant (*P* = .09). The addition of the motor and autonomy scale resulted in coefficients for ACT of −0.024 (95% CI: −0.046; −0.002) and −0.037 (95% CI: −0.060; −0.014), indicating indirect effects of 0.015 and 0.02, respectively (Table 3). A complete overview of all parameters of the mixed models is given in Appendix Table S2.

**TABLE 3:** Estimates for the mixed model regression parameters for the association between chemotherapy and HU

	Estimate^a^	95% Confidence interval	*P* value
Crude model^b^	−0.039	−0.062; −0.015	<.01
Adjusted model 1	−0.039	−0.062; −0.015	<.01
Adjusted model 2	−0.020	−0.044; 0.003	.09
Adjusted model 3	−0.024	−0.046; −0.002	.03
Adjusted model 4	−0.037	−0.060; −0.014	<.01

^a^ The difference in HU over time for patients treated with adjuvant treatment compared to no adjuvant treatment.

^b^ Crude mixed model which includes treatment, baseline measurement and time from start chemotherapy to follow‐up measurement. Model 1: Additionally corrected for age, gender and education level. Model 2: Additionally corrected for age, gender, education level and the sensory neuropathy scale. Model 3: Additionally corrected for age, gender, education level and the motor neuropathy scale. Model 4: Additionally corrected for age, gender, education level and the items of the autonomy neuropathy scale. Note that no summary score was calculated for the autonomy scale, due to the poor internal consistency.

### 3.4 Sensitivity analyses

Results for both sensitivity analyses are reported in Appendix Tables S3 and S4. In the analysis in which we corrected for potential confounding by indication using matching, we found a difference of −0.036 (−0.085; 0.012) in HU for the group with ACT compared to the group without ACT in a model corrected for age, sex and education level. Including the CIPN sensory, motor and autonomy scales resulted in effects of −0.031(−0.075; 0.013), −0.031 (−0.074; 0.013) and −0.045 (−0.089; −0.001). In the second sensitivity analysis in which we only included patients with a baseline measurement after surgery and before chemotherapy, we found an effect of −0.058 (−0.090; −0.027) in the adjusted model. After including the CIPN sensory, motor and autonomy scales the effect decreased to −0.036 (−0.067; −0.006), −0.044 (−0.072; −0.016) and −0.057 (−0.086; −0.028). The results of both sensitivity analyses were roughly in line with the findings in the main analyses. The slightly different findings for the impact of neuropathy in the matching analysis may be caused by the smaller sample size.

## 4 DISCUSSION

The primary aim of this study was to estimate the impact of ACT on HU over time in stage II and III colon cancer patients. Subsequently, we evaluated whether the impact of ACT on HU could be (partly) explained by its major side effect, peripheral neuropathy. We found a small but statistically significantly lower HU of −0.039 (95% CI: −0.062; −0.015) in patients who received ACT compared to patients without ACT in a mixed model adjusted for age, gender and education level. The difference in HU seems to be partly related to the sensory and motor neuropathy‐related side effects of ACT, given the decreases in ACT effect to −0.020 (95% CI: −0.044; 0.003) and −0.024 (95% CI: −0.046; −0.002), respectively. Overall, the differences in HU between patients with and without ACT are small, indicating that the effect of ACT on HU should have a minor role in the decision whether or not to prescribe ACT in stage II and III colon cancer patients.

There are only limited published data regarding the impact of ACT on stage II and III colon cancer patients' HU. In the study of Ness et al, a mean difference of 0.04 in average HU was reported between stage III patients who were treated with ACT and stage I/II patients who did not receive ACT, which is in line with our results.^11^ However, the absolute values for HU for both groups treated with and without ACT in the study by Ness et al are slightly (≈10%) lower compared to the values we found in the current study. There are a number of reasons that may explain the differences in absolute values. Firstly, Ness et al included rectal cancer patients as well, while our study was limited to colon cancer patients. Secondly, the data that we used in the current study were derived from a prospective cohort and contained repeated measurements, while Ness et al. used a retrospective study design. Thirdly, the study of Ness et al was conducted in 1999, and since that time diagnostic procedures have changed leading to stage migration.^24^ That is, a patient that was classified as stage II in the study of Ness et al would probably be classified as stage III in our study. Also other studies showed that the administration of ACT may decrease quality of life during the treatment period and in the months following chemotherapy.^2^, ^14^ A detailed comparison of the findings of these studies is hampered by the fact that these studies focused on specific domains of quality of life, such as self‐care, anxiety, mobility and pain, while our study focused on overall heath utility.

Several challenges should be taken into account in the interpretation of our results. In the PLCRC cohort, patients are included at different periods during their course of disease, resulting in patients without baseline or follow‐up measurement(s). At least one baseline measurement and one follow‐up measurement before 60 months after diagnosis per patient was required to include patients in the mixed‐model analyses.^17^ Although we were forced to exclude many measurements in the mixed model analyses due to this requirement, the results of the descriptive statistics (n = 859) and the mixed‐model analyses (n = 282) were in line with each other as are the baseline characteristics for both subsets. To illustrate, in the descriptive statistics we found a difference of 0.03 in HU between patients with and without ACT during chemotherapy and the first 12 months thereafter while we found a difference of 0.04 using a mixed model. Thus, taking into account relevant covariates, resulted in a slightly larger difference.

Although differences in HU were statistically significant in the mixed models, the absolute difference is small, which raises the question of its clinical significance. A measure to express the clinical significance of quality of life measurements is the minimally important difference (MID). The MID is defined as the smallest difference in score which patients perceive as beneficial.^25^ In the field of oncology MID scores in a range of 0.03 to 0.10 were reported for the EQ‐5D.^26^ Based on these MID scores, our results are on the boundary of clinical relevance. Therefore, our results indicate that the effect of ACT on HU should not play a major role in the decision whether or not to assign ACT.

Our mixed models were adjusted for the confounders age, sex and education level based on the previous literature.^14^ Given the role of the patients' clinical condition in the decision to prescribe ACT, it is likely that the performance status of the patient could also cause confounding in the investigated association. However, we were unable to include this covariate in the mixed model, which may have caused incomplete correction for confounding. Furthermore, we were not able to include stage in the mixed models due to the high correlation with chemotherapy prescription. To evaluate the potential impact of stage, we conducted two sensitivity analyses; a propensity score matching analysis and an analysis in which we only included patients for which the baseline measurement was taken during the time period “after surgery and before chemotherapy”. In both sensitivity analyses, the effect estimated in the model adjusted for age, sex and education level was comparable to the effect in the main analysis, which indicates a minor impact of stage on HU. During the collection of the PLCRC data, the Dutch guidelines for the duration of oxaliplatin‐based ACT in stage II and stage III colon cancer patients were adjusted from 6 to 3 months. Only a small subset of our population was intentionally treated for 3 months (approximately 15%), therefore we were unable to correct for treatment duration in the analyses. However, the SCOT trial, which compared 6 vs 3 months of ACT in stage II and III colon cancer patients, showed that the HU during ACT and the first months thereafter was significantly higher in patients who received 3 months ACT (HU 0.86) compared to 6 months ACT (HU 0.81).^15^ In the current study, we found an average HU of 0.83 for the group with ACT during and directly after chemotherapy, which is in line with average findings of the SCOT trial. Interestingly, despite the fact that planned treatment duration was 6 months for the majority of patients, the average actual treatment duration was 3.5 months. The main reason for this shorter treatment duration presumably was a premature discontinuation due to side effects.

In the current study, patients were mainly treated with CAPOX (57%) and capecitabine monotherapy (40%). Fluorouracil, leucovorin and oxaliplatin (FOLFOX) is rarely prescribed in the Netherlands (2% in the current study). Literature shows that the degree to which neuropathy side effects occur may differ between CAPOX and FOLFOX. To illustrate, the incidence of neuropathy was significantly lower for patients treated with CAPOX than for FOLFOX in the ACHIEVE trial from the start of the chemotherapy until 3 years thereafter.^27^ In contrast, the SCOT trial found similar incidences of neuropathy in the CAPOX and FOLFOX groups.^28^ Although the findings are contradictory, generalizing the results of the current study to countries where FOLFOX is the most commonly prescribed treatment, such as France, Italy, Canada and the United States, should be done with caution.

To our knowledge, this is the first study that evaluated the impact of peripheral neuropathy on the association between ACT and HU. Our results show that the sensory and motoric neuropathy‐related side effects influence the association between ACT and HU. When interpreting the results, it is important to consider that patients without ACT also scored higher than zero on the EORTC QLQ‐CIPN20 questionnaire, although the scores were significantly lower compared to the group that received ACT. The score >0 in the group without ACT is caused by questions in the EORTC QLQ‐CIPN20 that are not specifically related to neuropathy complaints, such as “Did you have cramps in your hands?” and “Did you have difficulty hearing?” These complaints also occur in elderly people without neuropathy‐related side effects. As a result, the role of neuropathy in the association between ACT and HU may be underestimated in the current study. This raises the question whether the EORTC QLQ‐CIPN20 is the optimal questionnaire to measure CIPN as it might be difficult to distinguish between patients with and without neuropathy complaints induced by chemotherapy.

The results of this study may serve as input for future cost‐effectiveness analyses. Prescribing adjuvant treatment in stage II and III colon cancer patients is a changing landscape and is referred to in the literature as a medical dilemma.^29^ Particularly in such complex treatment decisions, cost‐effectiveness studies are needed to support medical decision‐making.

In conclusion, a statistically significant but small decrease in HU of −0.039 during chemotherapy and the first 12 months thereafter was observed between stage II/III colon cancer patients treated with ACT compared to those without ACT. Given the small difference in HU between patients with and without ACT, the impact on HU should not play a major role in the treatment decision. The sensory and motor neuropathy‐related side effects of chemotherapy explained part of the association between chemotherapy and HU.

## CONFLICT OF INTERESTS

Gabrielle Jongeneel, Marjolein J. E. Greuter, Felice N. van Erning, Jos W. R. Twisk, Cornelis J. A. Punt and Veerle M. H. Coupé declared no potential conflict of interests. Miriam Koopman declared a potential financial conflict of interest by having an advisory role for Nordic Farma, Merck‐Serono, Pierre Fabre, Servier and institutional scientific grants from Bayer, Bristol Myers Squibb, Merck, Roche, Servier. Geraldine R. Vink declared potential financial conflicts of interests due to institutional grants for other research projects: Servier, Bayer, Sirtex, Merck, BMS, Lilly.

5

## ETHICS STATEMENT

The Prospective Dutch ColoRectal Cancer cohort received approval of the medical ethical review committee of Utrecht, The Netherlands (METC 12‐510), and is registered at Clinicaltrials.gov under NCT02070146. The study was performed in accordance with the Declaration of Helsinki. All participants have given their informed consent before inclusion.

## Supporting information


**Data S1**. Supporting Information

## Data Availability

Data of the Prospective Dutch ColoRectal Cancer cohort are available upon request after review by the scientific committee, and can be requested via de website www.plcrc.nl.
