# Supplementary material for: Longitudinal effects of adjuvant chemotherapy and related neuropathy on health utility in stage II and III colon cancer patients: A prospective cohort study
Source: Int J Cancer. 2021 Jan 25;148(11):2702–11. doi: 10.1002/ijc.33472 (PMC8048818; doi:10.1002/ijc.33472)
Supplement: Supplementary file 1 — Data S1. Supporting Information [file IJC-148-2702-s001.pdf]

Longitudinal effects of adjuvant chemotherapy and related neuropathy on health utility in stage II and III colon cancer patients: a prospective cohort study

Gabrielle Jongeneel, Marjolein JE Greuter, Felice N van Erning, Jos WR Twisk, Miriam Koopman, Cornelis JA Punt, Geraldine R Vink, Veerle MH Coupé

Table of contents

Appendix S1. EORTC QLQ – CIPN20 questionnaire. 3
Appendix Table S1. Differences in average health utility between stage II and III colon cancer patients. 4
Appendix Table S2. Parameter estimates for the mixed model analyses. 5
Appendix Table S3. Baseline characteristics of the PLCRC subsets which were used for the sensitivity analyses. 6
Appendix Table S4. Results of the sensitivity analyses. 7

Appendix S1. EORTC QLQ – CIPN20 questionnaire.

Sensory scale
1. Did you have tingling fingers or hands?
2. Did you have tingling toes or feet?
3. Did you have numbness in your fingers or hands?
4. Did you have numbness in your toes or feet?
5. Did you have shooting or burning pain in your fingers or hands?
6. Did you have shooting or burning pain in your toes or feet?
9. Did you have problems standing or walking because of difficulty feeling the ground under your feet?
10. Did you have difficulty distinguishing between hot and cold water?
18. Did you have difficulty hearing?
Motor scale
7. Did you have cramps in your hands?
8. Did you have cramps in your feet?
11. Did you have a problem holding a pen, which made writing difficult?
12. Did you have difficulty manipulating small objects with your fingers (for example, fastening small buttons)?
13. Did you have difficulty opening a jar or bottle because of weakness in your hands?
14. Did you have difficulty walking because your feet dropped downwards?
15. Did you have difficulty climbing stairs or getting up out of a chair because of weakness in your legs?
19. Only for those who driving cars, did you have difficulty using the pedals?[1]
Autonomy scale
16. Were you dizzy when standing up from a sitting or lying position?
17. Did you have blurred vision?
20. Only for males, did you have difficulty getting or maintaining an erection?[1]

[1] Note that this item was not taken into account in the analyses, because it only applies to a subgroup of the population.

Appendix Table S1. Differences in average health utility between stage II and III colon cancer patients.

	No adjuvant treatment					Adjuvant treatment				
Health utility, mean (sd)	Stage II	n	Stage III	n	p-value[1]	Stage II	n	Stage III	n	p-value[1]
Before surgery	0.86 (0.12)	89	0.80 (0.12)	21	0.052	0.82 (0.11)	10	0.86 (0.12)	79	0.766
After surgery, before start chemotherapy	0.86 (0.13)	79	0.81 (0.17)	21	0.326	0.79 (0.13)	8	0.81 (0.14)	106	0.714
During chemotherapy	0.87 (0.12)	185	0.83 (0.14)	41	0.187	0.86 (0.10)	21	0.82 (0.14)	185	0.310
First 12 months after chemotherapy	0.87 (0.11)	305	0.84 (0.11)	82	0.180	0.88 (0.09)	47	0.83 (0.12)	450	0.060
More than 12 months after chemotherapy	0.86 (0.11)	204	0.81 (0.10)	73	0.020	0.85 (0.10)	36	0.83 (0.12)	271	0.580

[1] Statistically significant differences for average health utility between stage II and stage III patients per treatment group (yes/no) and per defined time period were tested using the Mann-Whitney U test.

Abbreviations: sd = standard deviation

Appendix Table S2. Parameter estimates for the mixed model analyses

	Crude model[1]		Adjusted model 1		Adjusted model 2		Adjusted model 3		Adjusted model 4	
	Coefficient	95% CI	Coefficient	95% CI	Coefficient	95% CI	Coefficient	95% CI	Coefficient	95% CI
Chemotherapy (yes versus no)	-0.0388	-0.0623;-0.154	-0.0385	-0.0619;-0.0151	-0.0204	-0.0440;0.0032	-0.0241	-0.0462;-0.0020	-0.0367	-0.0597;-0.0137
Health utility at baseline	0.4997	0.4287;0.5707	0.4901	0.4195;0.5606	0.3683	0.2912;0.4455	0.3637	-0.2847;0.4426	0.4207	0.3446;0.4967
Time between start chemotherapy and follow-up measure	-1.19e-07	-3.36e-5;3.34e-5	7.03e-08	-3.32e-5;3.33e-5	1.68e-5	-1.68e-5;5.04e-5	1.02e-5	-2.31e-5;4.35e-5	2.54e-7	3.30e-5;3.35e-5
Gender (women versus men)	NA	NA	0.0096	-0.0149;0.0341	0.0092	-0.0143;0.0326	0.0141	-0.0090;0.0372	0.0120	-0.0122;0.0363
Age	NA	NA	-2.12e-06	-0.0012;0.0012	0.0009	-0.0002;0.0022	0.0009	-0.0003;0.0021	0.0004	-0.0008;0.0016
Education level										
Low (reference)										
Moderate	NA	NA	0.0267	-0.0028;0.0561	0.0168	-0.0114;0.0451	0.0258	-0.0017;0.0532	0.0259	-0.0030;0.0547
High	NA	NA	0.0422	0.0150;0.0693	0.0290	0.0028;0.0552	0.0385	0.0127;0.0642	0.0424	-0.0154;-0.0694
CIPN sensory scale baseline	NA	NA	NA	NA	-0.0015	-0.0028;-0.0002	NA	NA	NA	NA
CIPN sensory scale follow-up	NA	NA	NA	NA	-0.0017	-0.0023;-0.0011	NA	NA	NA	NA
CIPN motor scale baseline	NA	NA	NA	NA	NA	NA	-0.0002	-0.0012;0.0009	NA	NA
CIPN motor scale follow-up	NA	NA	NA	NA	NA	NA	-0.0023	-0.0030;-0.0016	NA	NA
CIPN autonomy scale baseline										
Item 16										
Item 17	NA	NA	NA	NA	NA	NA	NA	NA	-0.0028	-0.0240;0.0181
	NA	NA	NA	NA	NA	NA	NA	NA	-0.0291	-0.0429;-0.0152
CIPN autonomy scale follow-up										
Item 16	NA	NA	NA	NA	NA	NA	NA	NA	-0.0168	-0.0431;0.0094
Item 17	NA	NA	NA	NA	NA	NA	NA	NA	-0.0035	-0.0204;0.0134
Constant	0.4547	0.3935;0.5160	0.4289	0.3178;0.5399	0.4940	0.3862;0.6019	0.4893	0.3826;0.5959	0.5211	0.3989;0.6432

Abbreviations: CI=confidence interval, NA=not applicable, CIPN = Chemotherapy Induced Peripheral Neuropathy. [1]Crude mixed model which includes fixed effects for treatment, baseline measurement and time from start chemotherapy to follow-up measurement. Model 1: Additionally corrected for age, gender and education level. Model 2: Additionally corrected for age, gender, education level and the sensory neuropathy scale. Model 3: Additionally corrected for age, gender, education level and the motor neuropathy scale. Model 4: Additionally corrected for age, gender, education level and the items of the autonomy neuropathy scale. Note that no summary score was calculated for the autonomy scale, due to the poor internal consistency.

Appendix Table S3. Baseline characteristics of the PLCRC subsets which were used for the sensitivity analyses.

	Sensitivity analysis 1 Propensity score matching			Sensitivity analysis 2 Baseline measurement after surgery and before chemotherapy		
	All (n=76)	No chemotherapy (n=38)	Chemotherapy (n=38)	All (n=155)	No chemotherapy (n=79)	Chemotherapy (n=76)
Age, median (IQR)	67 (62-74)	69 (61-75)	67 (63-73)	67 (59-73)	69 (61-75)	65 (59-70)
Sex, number (%)						
Men	50 (66)	23 (61)	27 (71)	87 (56)	47 (59)	40 (53)
Women	26 (34)	15 (39)	11 (29)	68 (44)	32 (41)	36 (47)
Disease stage, number (%)						
II	20 (26)	10 (26)	10 (26)	69 (45)	65 (82)	4 (5)
III	56 (74)	28 (74)	28 (74)	86 (55)	14 (18)	72 (95)
Treatment regimen, number (%)						
Capecitabine monotherapy	NA	NA	18 (47)	NA	NA	25 (33)
CAPOX	NA	NA	20 (53)	NA	NA	49 (65)
FOLFOX	NA	NA	0	NA	NA	1 (1)
unknown	NA	NA	0	NA	NA	1 (1)
Education level, number (%)						
low	37 (49)	21 (55)	16 (42)	57 (37)	34 (43)	23 (31)
moderate	16 (21)	7 (19)	9 (24)	36 (23)	15 (19)	21 (28)
high	23 (30)	10 (26)	13 (34)	61 (40)	30 (38)	31 (41)

Abbreviations; IQR = interquartile range, NA = not applicable.

Appendix Table S4. Results of the sensitivity analyses

	Sensitivity analysis 1; propensity score matching (n=76)			Sensitivity analysis 2; baseline measurement in time period "after surgery and before chemotherapy" (n=155)		
	Estimate[1]	95% CI	P-value	Estimate[1]	95% CI	P-value
Crude model[2]	-0.031	-0.080;0.017	0.204	-0.053	-0.086;-0.020	0.002
Adjusted model 1	-0.036	-0.085;0.012	0.143	-0.058	-0.090;-0.027	0.000
Adjusted model 2	-0.031	-0.075;0.013	0.171	-0.036	-0.067;-0.006	0.019
Adjusted model 3	-0.031	-0.074;0.013	0.170	-0.044	-0.072;-0.016	0.002
Adjusted model 4	-0.045	-0.089;-0.001	0.047	-0.057	-0.086;-0.028	0.000

[1] The difference in health utility over time for patients treated with adjuvant treatment compared to no adjuvant treatment [2] Crude mixed model which includes treatment, baseline measurement and time from start chemotherapy to follow-up measurement. Model 1: Additionally corrected for age, gender and education level. Model 2: Additionally corrected for age, gender, education level and the sensory neuropathy scale. Model 3: Additionally corrected for age, gender, education level and the motor neuropathy scale. Model 4: Additionally corrected for age, gender, education level and the items of the autonomy neuropathy scale. Note that no summary score was calculated for the autonomy scale, due to the poor internal consistency.
